# Massive Hematochezia Secondary to Rectal Enema Injury: The Role of Sengstaken–Blakemore Tube for Hemostasis When Endoscopy Fails

**DOI:** 10.7759/cureus.13946

**Published:** 2021-03-17

**Authors:** Artsiom Klimko, Cristian G Tieranu, Carmen M Preda, Andrei O Olteanu, Elena M Ionescu

**Affiliations:** 1 Division of Physiology and Neuroscience, University of Medicine and Pharmacy “Carol Davila”, Bucharest, ROU; 2 Department of Gastroenterology, “Elias” Emergency University Hospital, Bucharest, ROU; 3 Department of Gastroenterology, University of Medicine and Pharmacy “Carol Davila”, Bucharest, ROU; 4 Department of Gastroenterology, Fundeni Clinical Institute, Bucharest, ROU

**Keywords:** lower gastrointestinal hemorrhage, balloon tamponade, rectal, enema, sengstaken–blakemore

## Abstract

In rare instances, rectal cleansing enemas may cause rectal injury, precipitating lower gastrointestinal hemorrhage (LGIH). In a subset of LGIH cases, the bleeding diathesis may fail to respond to traditional treatment modalities and can be life-threatening. We present a case of an 84-year-old female with cleansing enema induced rectal bleeding - she was a poor surgical candidate and due to lack of access to in-house interventional radiology teams, hemostasis was attempted with sui generis use of the Sengstaken-Blakemore tube. Our transanal application of the Sengstaken-Blakemore tube for the management of LGIH contributes further evidence supporting the use of balloon tamponade in achieving hemostasis in select patients when traditional therapeutic modalities are unavailable.

## Introduction

Acute overt lower gastrointestinal hemorrhage (LGIH) denotes blood loss distal to the ligament of Treitz, usually originating from the colon. LGIH generally warrants admission to the hospital - in most patients, LGIH resolves spontaneously or exclusively requires conservative therapy with favorable outcomes. However, in instances where there is a delay in diagnosis and management or the presence of co-morbidities, LGIH may be life-threatening. In-hospital mortality rates range from 2.5% to 4.0% and up to 19% of patients experience an episode of rebleeding within one year [1,2]. Risk factors for mortality in hospitalized patients with LGIH include age greater than 70 years, intestinal ischemia, a Charlson comorbidity score of 2 or more, and coagulopathy [2,3]. Variable presentation of LGIH and inherent problems in localizing the source of LGIH may preclude effective treatment, especially when standard therapy fails to achieve hemostasis.

LGIH can be treated with a variety of treatment modalities and typically, the therapeutic approach is tailored to the underlying cause of the bleeding stigmata. After the bleeding site has been identified, hemostasis is typically attempted endoscopically with mechanical clips, submucosal injection of vasoconstricting agents, and bipolar coagulation. Angiography is an alternative technique and relies on a tagged red blood cell scan to identify the bleeding vessel and its subsequent embolization. If angiographic control fails and other imaging modalities (e.g. scintigraphy) are unsuccessful in localizing the bleeding site, segmental or subtotal colectomy may be required.

The primary application of tamponade devices (e.g. Sengstaken-Blakemore or Minnesota tubes) is management of esophageal variceal hemorrhage, although their use has been documented in occluding any major vascular or solid organ injury that is inaccessible [4]. In such instances, tamponade devices are used temporarily (often less than six hours) in patients with high shock indices and achieve hemostasis of bleeding structures - this measure is successful in up to 93% of cases [4]. Herein we present a patient who developed life-threatening LGIH - she was a poor surgical candidate and a balloon tamponade device was successfully utilized to achieve hemostasis.

## Case presentation

An 84-year-old female was admitted for staging a vulvar neoplasm in the Oncology Department of “Elias” Emergency University Hospital. The patient suffered from a three-day bout of constipation - treatment was attempted with a cleansing enema, which precipitated a massive LGIH and required gastroenterology consultation. She presented with multiple high-volume, bloody stools, a mean arterial blood pressure of 65 mmHg, and rapidly declining hemoglobin (from 8.4 g/dL at admission to 6.0 g/dL at the moment of the gastroenterology consult).

**Figure 1:**
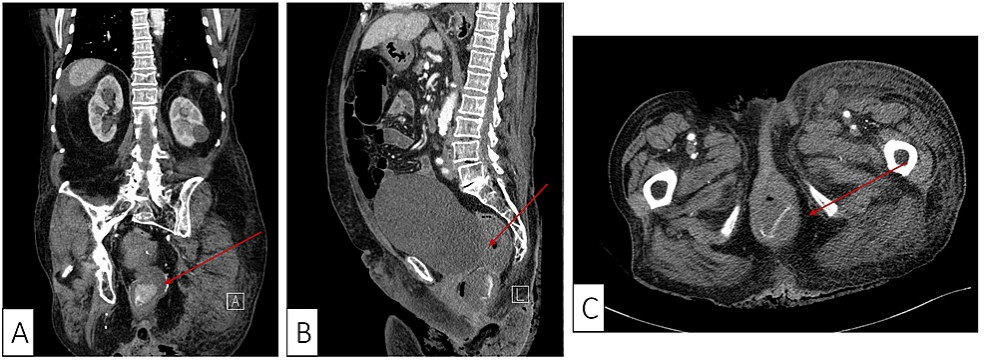
Contrast-enhanced computed tomography sections (A – coronal, B – sagittal, C – transverse) showing active contrast solution leakage in the rectal cavity (red arrow).

The patient was transferred to the intensive care unit (ICU) for stabilization. We initiated urgent fluid resuscitation with 2,000 mL of crystalloid fluids and three units of packed red blood cells. The arterial blood gas revealed a metabolic acidosis (pH 7.28), hypocapnia, elevated lactate (5.6 mmol/L), and a high base deficit (-12 mEq/L). An emergency computed tomography (CT) was performed in order to rapidly identify the source of the bleed and adapt further management based on the site of injury. CT showed a distended rectum with fluid content and leakage of intravenous contrast solution into the rectal cavity during the late arterial phase (Figures 1A-1C).

Without bowel preparation, an emergency flexible sigmoidoscopy was performed and showed fresh blood in the rectal cavity - an “oozing” active bleed appeared to be in close vicinity (6-7 cm) to the anal verge. Unfortunately, the lack of precursive rectal cleansing impeded identification of the lesion and its endoscopic hemostasis. Massive GI bleeding with hemodynamic deterioration and the lack of in-house interventional radiologists presented a significant clinical challenge.

**Figure 2:**
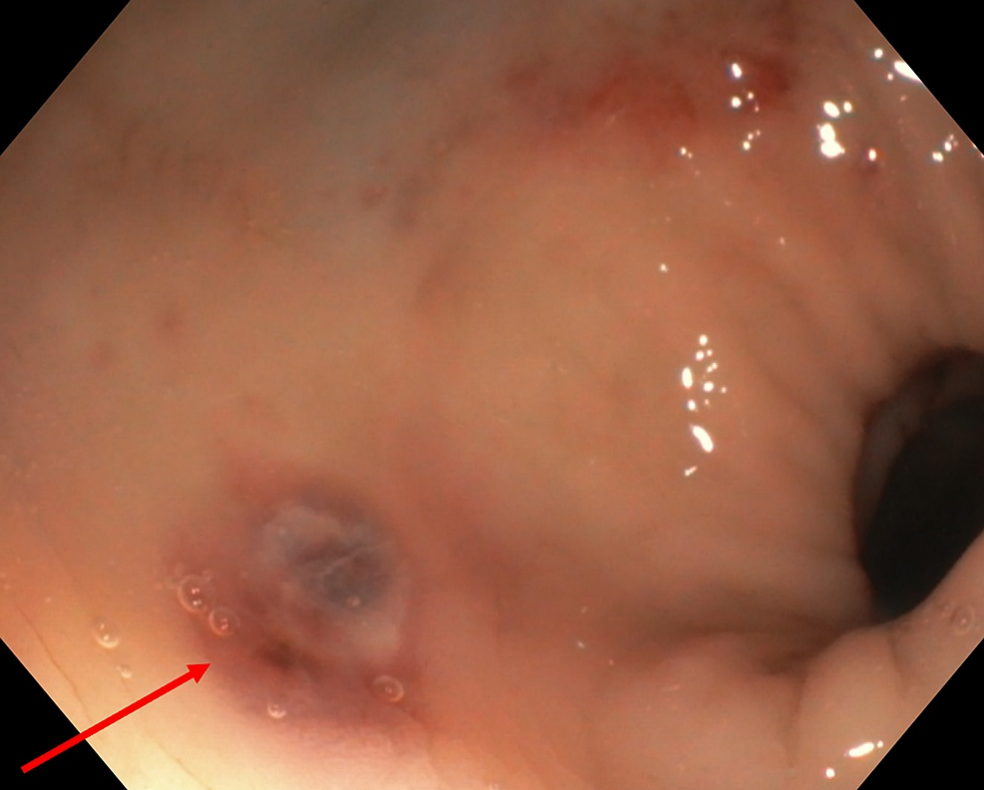
Traumatic rectal lesion (red arrow) with vascular appearance seen on repeat sigmoidoscopy after application of balloon tamponade.

After an emergency multidisciplinary discussion with the surgical team, hemostasis was attempted via transanal placement of a Sengstaken-Blakemore tube with intrarectal inflation of the gastric balloon. The patient’s informed consent was obtained before the procedure. The gastric balloon was inflated with 180 mL of air and the patient’s visceral pain was used to guide the upper limit of inflation. Local evolution was monitored using the drainage volume as an indirect marker. The drainage stopped four hours after the procedure, the patient’s hemodynamic status improved, and it was deemed safe to withdraw the vasopressors. At 12 hours after the procedure, there was no drainage and the patient was clinically stable. The Sengstaken-Blakemore tube was removed and another flexible sigmoidoscopy was performed, showing a traumatic lesion of the rectum 8 cm from the anal verge with recent bleeding stigmata (Figure 2). The patient was maintained on a clear liquid diet for another 24 hours, and a normal diet thereafter without relapse of the lower GI bleeding. The rest of her hospital admission was uneventful and the patient was discharged seven days later.

## Discussion

In this case report, we presented a patient with an active post traumatic rectal bleeding, without perforation or hematoma, induced by a cleansing enema - endoscopic attempts at achieving hemostasis failed and a novel technique using intrarectally inserted Sengstaken-Blakemore gastric balloon tamponade controlled the bleeding. Rectal enema injuries have been reported in the literature ranging from hematomas in patients on antiplatelet/anticoagulant treatment to rectal perforation of different stages [5,6]. There have been several reports describing the role of balloon tamponade for lower GI bleeding of different causes, all of them showing good efficacy [7-13].

As compared to esophageal variceal bleeding, where balloon tamponade is well established as a therapeutic option, its use in LGIH has been mostly reported in case reports across literature, thus precluding it from being widely used for any other indication than variceal bleeding. In order to further explore the application of tamponade devices to manage LGIH, a search of the PubMed and Scopus databases was conducted. A set of keywords were used to identify relevant articles and per our selection strategy, seven relevant case reports were found - a total of eight patients were evaluated and our findings are presented in Table 1.

**Table 1:** List of case reports which utilized a balloon tamponade device to manage intractable lower gastrointestinal hemorrhage. CT: computed tomography.

Author and year	Gender and age	Location of hemorrhage	Etiology of hemorrhage	Type of balloon used and tamponade time	Reason for use of tamponade
Our case	Female, 84	Anterior rectum, 8 cm from anal verge	Cleansing enema	Sengstaken-Blakemore tube (180 mL); 12 hours	Poor surgical candidate; interventional radiology teams were unavailable in-house
Fadel et al., 2020 [7]	Male, 75	Anterior rectum, 4 cm from anal verge	Idiopathic	Sengstaken-Blakemore tube (100 mL); 36 hours	Poor surgical candidate; source of hemorrhage not detected by CT angiography
Neeki et al., 2019 [8]	Male, 76	Ulcerated mucosa at dentate line	Idiopathic	Minnesota tube (200 mL gastric balloon, 300 mL esophageal balloon); 24 hours	Poor surgical candidate; due to late hour of presentation, interventional radiology teams were unavailable in-house
Michopoulou et al., 2013 [9]	Female, 64	Anterior rectum, 15 cm from anal verge	Rectal biopsy; complicated by pseudomembranous colitis	Sengstaken-Blakemore tube (250 mL); deflated at 48 h, removed at 60 hours	Poor surgical candidate; interventional radiology teams were unavailable in-house
Marshall et al., 2007 [10]	Male, 54	Stapled ileorectal anastomosis	Complication of total colectomy	Minnesota tube (200 mL gastric balloon); 60 hours	Intractable bleeding following colectomy
Cho et al., 2006 [11]	Male, 51	Internal rectal varices	Possibly precipitated by placement of a transjugular intrahepatic portosystemic shunt	Minnesota tube (200 mL gastric balloon); 24 hours	Intractable variceal bleeding
McGuinness et al., 2004 [12]	Male, 65	Resected polyp in the posterior mid-low rectum	Transanal polypectomy	Minnesota tube (50 mL gastric balloon, 100 mL esophageal balloon); deflated after 12 hours, removed after 24 hours	Intractable bleeding that failed standard therapy
Roy et al., 1996 [13]	Female, 75	Lower third of rectum	Angiodysplasia	Sengstaken-Blakemore tube (350 mL); 48 hours	Intractable bleeding that failed standard therapy

The average age of the patients was 68 (range 54-84), and of the eight patients, five were male and three were female. The etiology of the LGIH was diverse and half of the patients were treated with a Minnesota tube, while the other half with a Sengstaken-Blakemore tube. The case by Marshall et al., was particularly striking as it involved a patient who presented with LGIH secondary to an ulcerated arterial vessel in the wall of a diverticulum [10]. Intractable LGIH was managed with total colectomy with ileorectal anastomosis, which again presented with severe bleeding. A Minnesota tube was inserted via the ileostomy, inflated to tamponade the bleeding stigmata - it was kept inflated for 60 hours, which is the longest reported time in our review.

The clinical picture of the patients was dominated by co-morbidities, such as decompensated liver cirrhosis, chronic kidney disease, or severe chronic obstructive pulmonary disease, which made them poor candidates for emergency surgery. The decision to use a balloon tamponade device was typically taken after typical treatment measures, namely, electrocautery, local adrenaline injections, resolution clips or other types of suture ligations were not successful. Furthermore, angiography was either unable to detect the bleeding site or an in-house interventional radiology team was unavailable to attempt radiological embolization.

The fragility of colonic mucosa increases the likelihood of hemorrhage, as compared to esophageal mucosa. Excessive pressure can lead to ulceration and pressure necrosis of the compressed visceral wall, which must be balanced against underinflation which can predispose to migration and ultimately, failure of the tamponade. Although no iatrogenic complications were reported in the reviewed literature, strategies to reduce the risk of such injuries warrant attention. Four studies put forth specific recommendations for both the Sengstaken-Blakemore and Minnesota tubes and are summarized in Table 2.

**Table 2:** Proposed strategies to avoid iatrogenic complications when using balloon tamponade devices to manage intractable lower gastrointestinal hemorrhage.

Author and year	Type of tamponade device used	Proposed strategy to avoid iatrogenic complications
Michopoulou et al., 2013 [9]	Sengstaken-Blakemore	Conduct five-minute deflations every six hours
Marshall et al., 2007 [10]	Minnesota tube	Conduct five-minute deflations every 12 hours
McGuinness et al., 2004 [12]	Minnesota tube	Avoid inflation for longer than 12 hours and deflate if visceral pain develops
Roy et al., 1996 [13]	Sengstaken-Blakemore	Conduct five-minute deflations every hour

The use of the Sengstaken-Blakemore tube in esophageal variceal bleeding is a temporizing, emergent rescue measure and risks associated with using mechanical compression to arrest hemorrhage must be evaluated within that frame of reference. The most common causes of death in patients treated with the Sengstaken-Blakemore tube for recalcitrant esophageal variceal bleeding are failure of hemostasis (42.9%), rebleeding (17.9%), and esophageal perforation (14.3%) [14]. As esophageal perforation is the only addressable cause of lethal complications, any conditions that increase the risk of perforation (esophageal or gastric surgery or esophageal strictures) are the only relative contraindications to the use of tamponade devices.

Furthermore, patients who require tamponade devices for managing variceal hemorrhage are, by definition, end-stage cirrhotics with high 30-day mortality rates (42.4%) after bleeding diatheses requiring the Sengstaken-Blakemore tube [14]. Therefore, when comparing patient cohorts that were treated with pressure tamponade for variceal versus LGIH, only limited conclusions regarding the limitations of this treatment modality in LGIH management can be drawn.

## Conclusions

Balloon tamponade represents a simple, rapid, and effective technique to manage recalcitrant LGIH when conventional treatments fail. Our findings suggest that such an unconventional application of balloon tamponade may be safe and provide a sometimes-critical opportunity to stabilize patients with elevated shock indices. This may be especially relevant for patients who are poor surgical candidates and in whom angiographic and endoscopic therapy either failed or was unavailable. Recommendations to prevent iatrogenic trauma include using the patient's visceral pain as a surrogate for insufflation volumes, applying short and regular intervals of deflation, and limiting the in-place maintenance time of the tamponade device.
